# Performance of Bio-Rad and Limiting Antigen Avidity Assays in Detecting Recent HIV Infections Using the Quebec Primary HIV-1 Infection Cohort

**DOI:** 10.1371/journal.pone.0156023

**Published:** 2016-05-25

**Authors:** Bouchra Serhir, Denis Hamel, Florence Doualla-Bell, Jean Pierre Routy, Sylvie-Nancy Beaulac, Mario Legault, Micheline Fauvel, Cécile Tremblay, Quebec Primary HIV infection study group

**Affiliations:** 1 Laboratoire de santé publique du Québec/Institut national de santé publique du Québec, Sainte-Anne-de-Bellevue, Québec, Canada; 2 Unité de surveillance des maladies chroniques et de leur déterminants/Institut national de santé publique du Québec, Québec, Québec, Canada; 3 Department of Medicine, Division of Experimental Medicine, McGill University, Montreal, Quebec, Canada; 4 Chronic Viral Illness Service and Division of Hematology, McGill University Health Centre, Montreal, Quebec, Canada; 5 Réseau SIDA Maladies infectieuses, Fonds de la recherche du Québec-Santé Montréal, Montréal, Québec, Canada; 6 Département de microbiologie, infectiologie et immunologie, Faculté de médecine, Université de Montréal, Montréal, Québec, Canada; University of California, San Francisco, UNITED STATES

## Abstract

**Background:**

Accurate and practical biologic tools to estimate HIV incidence is crucial to better monitor the epidemic and evaluate the effectiveness of HIV prevention and treatment programs.

**Methods:**

We evaluated two avidity assays to measure recent HIV infection: the Sedia HIV-1 LAg-Avidity EIA (Sedia Biosciences, Portland) and the Centers for Disease Control and Prevention (CDC)-modified Bio-Rad-Avidity assay (Bio-Rad Laboratories, Mississauga, ON). Longitudinal specimens (n = 473) obtained from 123 treatment-naive seroconverted individuals enrolled in the Primary HIV-1 Infection (PHI) cohort of Quebec were used to determine the average time an individual is considered to be recently infected (mean duration of recent infection; MDRI), for the two avidity assays alone and in combination using a nonparametric survival method analysis. A total of 420 specimens from individuals with established HIV infection (90 individuals from the PHI cohort of Quebec and 330 individuals from the Laboratoire de santé publique du Quebec (LSPQ) serobank) were also tested to investigate false recency rate (FRR).

**Results:**

The CDC-modified Bio-Rad-Avidity gave an estimated MDRI of 234 days (95% CI 220–249) at the avidity index cutoff of 30% while the Sedia-LAg-Avidity assay gave an estimated MDRI of 120 days (95% CI 109–132) at the normalized optical density (ODn) cutoff of 1.5. The FRR among individuals with established HIV infection was 10.2% (7.5%-13.5%) with the CDC-modified Bio-Rad-Avidity assay as compared to 6.0% (3.9%-8.7%) with the Sedia-LAg-Avidity assay. When optimizing a multiassay algorithm (MAA) that includes sequentially the CDC-modified Bio-Rad-Avidity assay then the Sedia-LAg-Avidity assay EIA (avidity index/ODn: 30%/1.7), the MDRI was 136 days (95% CI 123–148) and the FRR, 3.3% (95% CI 1.8–5.6).

**Conclusion:**

Multiassay algorithms that include the CDC-modified Bio-Rad-Avidity assay and the Sedia-LAg-Avidity assay performed better than each avidity assay alone. Such 2-assay algorithm that starts with the CDC-modified Bio-Rad-Avidity assay followed by the Sedia-LAg-Avidity assay allowed a better classification of HIV-1 infections.

## Introduction

Measuring the incidence rate of new HIV infections in a given population [1], is crucial to monitor the evolution of the epidemic, to identify populations at risk of acquiring HIV and to measure the impact of intervention programs [2–4]. In 2008, the World Health Organization (WHO) coordinated a Technical Working Group on incidence assays in order to improve their accuracy and develop guidelines for their proper use. This group published their recommendations on essential conditions for improving estimates of incidence, including the determination of the mean duration of recent infection (MDRI) and calculating the false recency rate (FRR) [5]. Although, there was no consensus on how to measure incidence, HIV incidence assays were considered as an optimal tool that should be developed and implemented for accurately measuring changes in HIV incidence. These recommendations aimed at identifying early HIV infection, in order to improve access to treatment and care as well as to prevent secondary transmission.

Different laboratory assays have been developed and implemented, most often using cross-sectional population analysis to estimate MDRI of HIV infection [6–15]. Some adjustments, due to an over-estimation of incidence, were implemented to the currently used BED-capture-enzyme immunoassay [16] whereas new promising avidity-based assays have been developed, including the CDC-modified-Bio-Rad Avidity and the Sedia-LAg-Avidity assays [17–19]. These avidity assays measure the strength of the bond between HIV viral proteins and HIV-specific antibodies. Indeed, low avidity-antibodies occurring early during the course of infection are indicative of recent infection. Although the avidity parameters are likely to be accurate in classifying recent infection, they require a better standardization and definition of threshold cutoff and mean duration of recent infection [12]. The main challenges for estimating HIV incidence remain the misclassification of long-standing infections and the MDRI estimation, corresponding to the average time an individual has been considered to be recently infected [4, 20]. In order to provide with more accurate HIV incidence estimates, several multiassay algorithms (MAAs) were proposed that include multiple serologic assays in absence or presence of biomarkers such as CD4^+^ T-cell count or viral load [21–23].

The Quebec HIV surveillance program does not differentiate between recent and long-standing infections. In order to detect and to monitor recent infections among populations at risk in Quebec, we evaluated the performance of two avidity-based assays, the CDC-modified Bio-Rad-Avidity assay [18] and the more recently developed limiting-antigen avidity assay, the Sedia-LAg-Avidity assay [17, 19], alone or in combination, using longitudinal samples from the well characterized PHI cohort of Quebec [24].

## Materials and Methods

### Ethics statement

All work was conducted in accordance with the Declaration of Helsinki in terms of informed consent and approval by the appropriate institutional review board. Written informed consent was obtained from individuals in the PHI cohort of Quebec. The ethical approval was given and renewed each year by the McGill University Health Centre Research Ethic Board and le Comité d'éthique et de la recherche des Centres hospitaliers affiliés à l'Université de Montréal. The ethical approval for the AIDS Research Network Private Medical Clinics was given and renewed each year by Veritas-Ethica Clinical Research Inc., an independent Review Board.

### Longitudinal specimens from the PHI cohort of Quebec

Longitudinal (n = 577) and long-standing infection (n = 90; drawn>14 months post infection) specimens were collected within the PHI cohort from which each participant (n = 240) was confirmed to be recently infected with HIV (<6 months after seroconversion), between 1997 and 2011. Of the 150 individuals from whom 577 longitudinal specimens were available, 123 were treatment-naive while 27 were treatment-experienced. Three to four serial specimens per participant, obtained within the first year post infection, were used to calculate MDRI in different sample sets, including treatment-naive patients (n = 123) and both treatment-naive and treatment-experienced patients (n = 150). The 90 single long-standing infection specimens from the PHI cohort were added to cross-sectional LSPQ serobank specimens (n = 330) in order to estimate the FRR.

All participants provided informed consent for blood collection and resistance testing and completed standardized nurse-administered questionnaires describing risk factors, mode of transmission, age, and treatment status [24–26]. Viral loads, CD4^+^ T-cell counts, antiretroviral treatment (ART) and clinical epidemiological data were also collected. The estimated date of infection, for each participant, was well defined based on the following criteria: 1) detectable HIV-RNA and negative EIA; and 2) documented seroconversion by western blot (positive WB).

#### Cross-sectional HIV-1 positive specimens

Cross-sectional HIV-1 diagnostic specimens were obtained from the LSPQ serobank collection. The LSPQ is the Public health laboratory of the province of Québec where confirmation of HIV infection is performed. All LSPQ specimens used in this study are residual sera collected for routine diagnostic purpose between 1995 and 2012. The serum specimens were all repeatedly reactive using a screening HIV 1, 2 EIA assay and then confirmed positive for HIV-1 using the Health Canada approved GS HIV-1 WB (Bio-Rad Laboratories, Mississauga, ON). A total of 330 specimens, obtained from individuals with established HIV infection, were used for FRR estimation. In this study, established infection was defined as follows: specimen obtained from an individual who was previously confirmed positive by WB for over 24 months.

### CDC-modified Bio-Rad-Avidity assay [18]

Modifications were brought to the commercial Genetics Systems HIV-1/HIV-2 Plus O EIA (Bio-Rad Laboratories, Mississauga, ON). Briefly, specimens are diluted 1:10 in a cold specimen diluent, transferred to two wells of the test plate and incubated for one hour at 4°C. HIV antibodies present in the sample bind to the antigen coated solid phase of the plate. Wells are then treated in parallel with either 0.1M Diethylamine (DEA) dissociation agent or 1x wash buffer. Incidence and prevalence internal controls were used in each test run. An assay is considered valid if the incidence control is below 20%, and the prevalence control higher than 80%. An avidity index (AI) is calculated from both OD values (OD (DEA)/OD (wash buffer) x 100) for each sample. Samples with an AI between 20% and 50% were retested in duplicate, and the average of both confirmatory avidity results was considered as final AI. Specimens with an AI ≤ 30% were classified as recent infection. Based on this avidity protocol, the MDRI (according to Masciotra and Owen, personal communication) is 220 days for infections with subtype B.

### Sedia-LAg-Avidity assay [17]

The Sedia-LAg-Avidity assay (Sedia^™^ HIV-1 LAg Avidity EIA; Sedia Biosciences Corporation, Portland, OR, USA) is a new commercially available antibody-single well based incidence assay. The antigens used are recombinant proteins containing HIV-1 immunodominant region of gp41. Using 0.1 M citrate buffer as a dissociation agent, antibody avidity is measured as a normalized optical density (ODn) value. After a first screening assay, specimens with an ODn value > 2.0 are classified as high avidity specimens; those with an ODn ≤ 2.0 are retested in triplicate in a confirmatory assay. In the confirmatory assay, specimens with a median ODn ≤ 1.5 are classified as recent while those > 1.5 are classified as long-standing. According to the manufacturer, the estimated MDRI, at a cutoff of 1.5, is 130 days [27].

### Determination of the mean duration of recent infection of infection

For both avidity assays, the nonparametric survival method analysis accounting for interval-censored data was used to estimate MDRI [28, 29]. MDRI is equivalent to the area under the survival curve by assuming, for the longest observed subject, that the recent infection occurred at the latest observed time. We ran the new ICLIFETEST SAS procedure to derive the appropriate survival curve [28]. The MDRI for the MAA was calculated at varying cutoff values for the Sedia-LAg-Avidity (1.1, 1.2, 1.3, 1.4, 1.5, 1.6, 1.7, 1.8, 1.9 and 2.0) and the CDC-modified-Bio-Rad-Avidity (25%, 30%, 35% and 40%) assays. Individuals from the PHI cohort, from whom three to four serial specimens were available, were used to estimate the MDRI values. Specimens were collected at different times after exposure up to 400 days.

### Determination of false recent rate

Our goal was to achieve a FRR less than 5% (confidence interval of 95%). Using the exact distribution of these probabilities (binomial), a sample size of 420 long-standing specimens from 420 individuals presenting with over 14 months post infection, was selected for FRR calculation. Individuals selected for the FRR were distinct from those selected for the calculation of the MDRI. Statistical power and sample size calculations were performed using PASS software. Statistical analysis was performed using SAS 9.4 software (SAS Institute, NC).

## Results

### Characteristics of individuals and samples used in the study

A total of 997 specimens from individuals with well documented HIV infection was tested. Table 1 describes the characteristics of the samples from the PHI cohort (n = 667) and the LSPQ serobank collection (n = 330).

**Table 1:** Characteristics of individuals and samples used for analysis.

Characteristics	HIV-1 PHI cohort (n = 667)				LSPQ serobank (n = 330)
	MDRI (All)	MDRI (naive)	MDRI (treated)	FRR	FRR
**No. of samples**	577	473	104	90	330
**No. of unique individuals**	150	123	27	90	330
**Age, mean [range]**	36.8 [19–59]	36.3 [19–58]	39.6 [20–59]	35.1 [18–58]	ND
**No. of samples per individual**	3–4	3–4	3–4	1	1
**Male sex (% of individuals)**	144 (96%)	118 (96%)	26 (96%)	86 (95.5%)	ND
**Ethnicity**					ND
White/Black	139/5	113/5	26/0	83/3	
Hispanic	6	5	1	2	
Asian	0	0	0	2	
Risk factor for HIV acquisition					
MSM/IDU/heterosexuals	117/28/5	96/22/5	21/6/0	66/18/6	ND
**Baseline CD4+ T-cell count (cells/mm**^**3**^**): No. of individuals [median, range]**					
> 500	74 [513, 510–1413]	65 [670, 510–1413]	9 [610, 516–822]	36 [630, 501–1470]	
301–500	62 [445, 310–500]	51 [445, 310–500]	11 [410, 330–484]	40 [402, 309–500]	
201–300	9 [289, 210–300]	5 [289, 220–290]	4 [255, 210–300]	9 [278, 249–300]	
51–200	4 [165.5, 68–200]	1 [191]	3 [140, 68–200]	4 [185, 140–200]	
≤ 50	0	0	0	1 [30]	
Missing	1	1	0	0	
**HIV load (log10 copies/mL): No. of individuals [median, range]**					ND
>4,7	67 [5.1, 4.7–6.7]	46 [5.0, 4.7–6.5]	21 [5.2, 4.8–6.7]	42 [5.1, 4.7–6.0]	
>4,0 to 4,7	40 [4.4, 4.0–4.7]	36 [4.4, 4.0–4.7]	4 [4.4, 4.1–4.5]	28 [4.3, 4.0–4.7]	
>2,6 to 4,0	34 [3.6, 2.9–4.0]	32 [3.6, 2.9–4.0]	2 [3.7, 3.0–4.0]	18 [3.5, 2.8–3.9]	
≤2,6	9 [2.3, 1.7–2.6]	9 [2.3, 1.7–2.6]		2 [2.0, 2.0–3.1]	
**No. of individuals receiving ART: [median, range]**				ND	ND
Before enrolment	0	0	0		
During the study					
ART 0–30 days	2 [14.5, 0–29]	0	2 [14.5, 0–29]		
ART 31–90 days	0	0	0		
ART 91–170 days	8 [127, 99–163]	0	8 [127, 99–163]		
ART 171–400 days	17 [303, 181–364]	0	17 [303, 181–364]		

Abbreviations. FRQ-S: Fonds de la recherche du Québec en Santé; MSM: Men who have sex with man; ART: antiretroviral therapy. Subgroups of individuals involved in calculating MDRI: mean duration of recent infection; FRR: false recent rate, ND: not determined; No: number.

Samples from the PHI cohort were collected between 1997 and 2011, near the time of seroconversion up to 400 days post-seroconversion (150 individuals, n = 577 specimens) and over 14 months post-seroconversion (90 individuals, 90 specimens). For each of these individuals, epidemiological data and laboratory data, including CD4^+^ T-cell count, plasma HIV viral load and antiretroviral treatment (ART) were available. Of the 240 PHI individuals, 230 (95.8%) were male, 222 (92.5%) were Caucasians, and 238 (99%) were infected with HIV-1 subtype B. The two individuals infected with HIV-1 non-B subtypes were infected with HIV-1 subtype A/D and HIV-1 subtype F, respectively. In addition, 183 individuals were men who have sex with man (MSM, 76.2%), 46 intravenous drug users (IDU, 19%), and 11 heterosexuals (4.5%).

Among the 150 individuals from the PHI cohort who participated to the MDRI estimation, four individuals infected with HIV-1 subtype B presented with a CD4^+^ T-cell counts less than 200 cells/mm^3^. Three of them, treated with ARVs at days 101, 119 and 259 post-seroconversion, were Caucasians, infected with HIV-1 subtype B, and exhibited a viral load > 5.7 log_10_ copies/mL. The other individual is a Caucasian IDU man who had a viral load of 6.5 log_10_ copies/mL.

Nine (6%) of the 150 MDRI seroconverters exhibited a viral load lower than 2.6 log_10_ copies/mL (median: 2.3, range 1.7–2.6). All were ART naive and had a CD4^+^ count higher than 450 cells/mm^3^ (median: 710, range: 468–940). The nine participants were diagnosed between 1997 and 2010; seven were MSM, one IDU and one heterosexual; seven were Caucasians and two black, and all were known to be infected with HIV-1 subtype B. The two individuals infected with HIV-1 non-B subtype were male, MSM and treatment-naive. The one infected with HIV-1 subtype A/D presented with a CD4^+^ T-cell count of 560 cells/mm^3^ and a viral load of 3.5 log_10_ copies/mL. The patient infected with HIV-1 subtype F exhibited a viral load of 3.6 log_10_ copies/mL and a CD4^+^ T-cell count of 480 cells/mm^3^.

Individual’s clinical characteristics such as CD4^+^ T-cell count, viral load and ART exposure were not documented for the LSPQ serobank specimens.

### Comparison of antibody-avidity kinetics exhibited by the CDC-modified Bio-Rad-Avidity and the Sedia-LAg-Avidity assays

**Fig 1:**
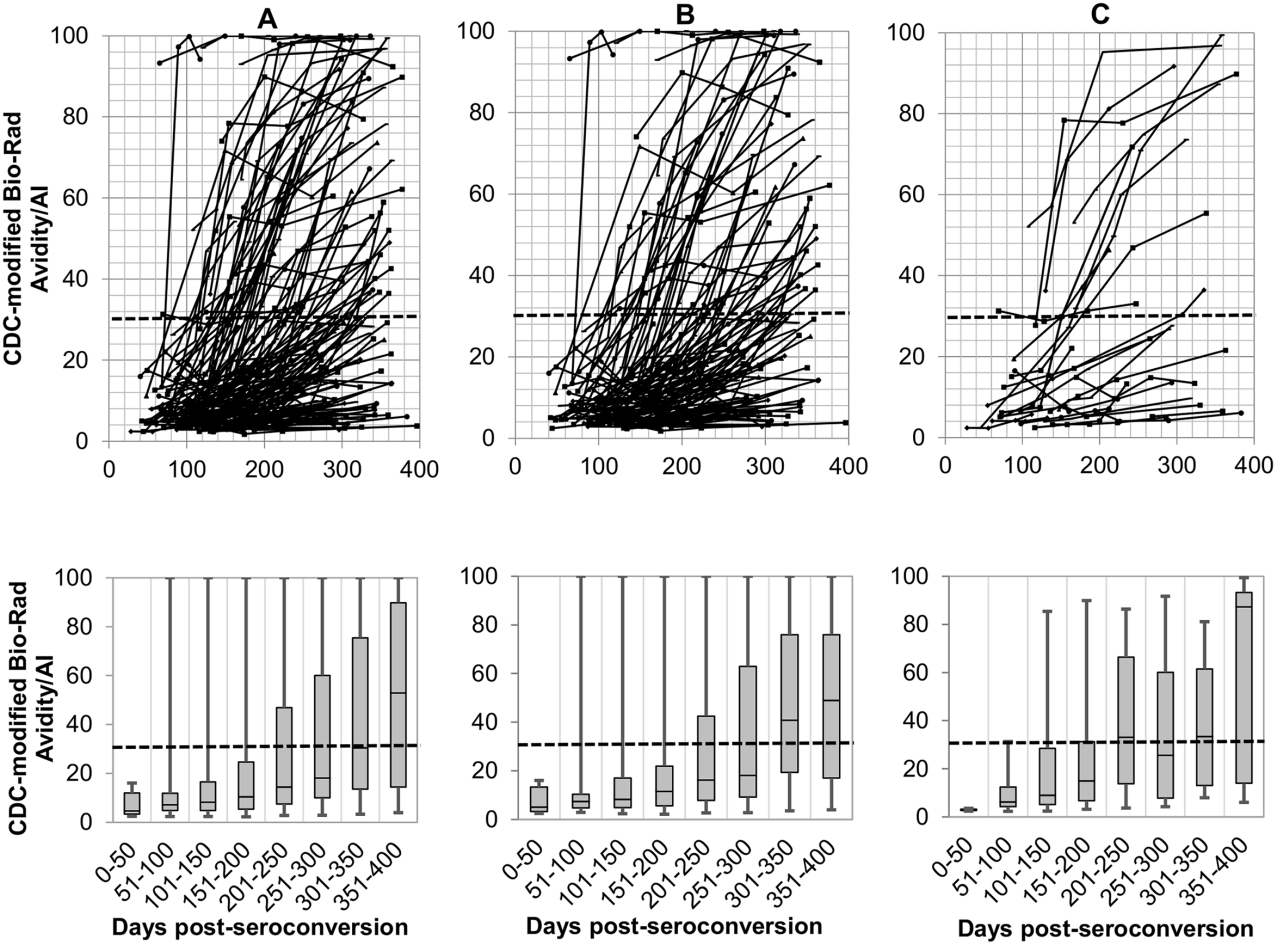
HIV antibody kinetics (up) and box-and-whisker plots showing median of AI measurements at 50 days intervals (bottom) of the all 150 seroconverting individuals of the PHI cohort of Québec (A), the 123 treatment-naive patients of the PHI cohort of Québec (B), the individuals of the PHI cohort of Québec treated between 0 and 400 days post-seroconversion (C), using the CDC-modified Bio-Rad-Avidity assay. The ‘recent/long-standing’ infection cutoff value is shown by the horizontal dashed line. AI: avidity index.

**Fig 2:**
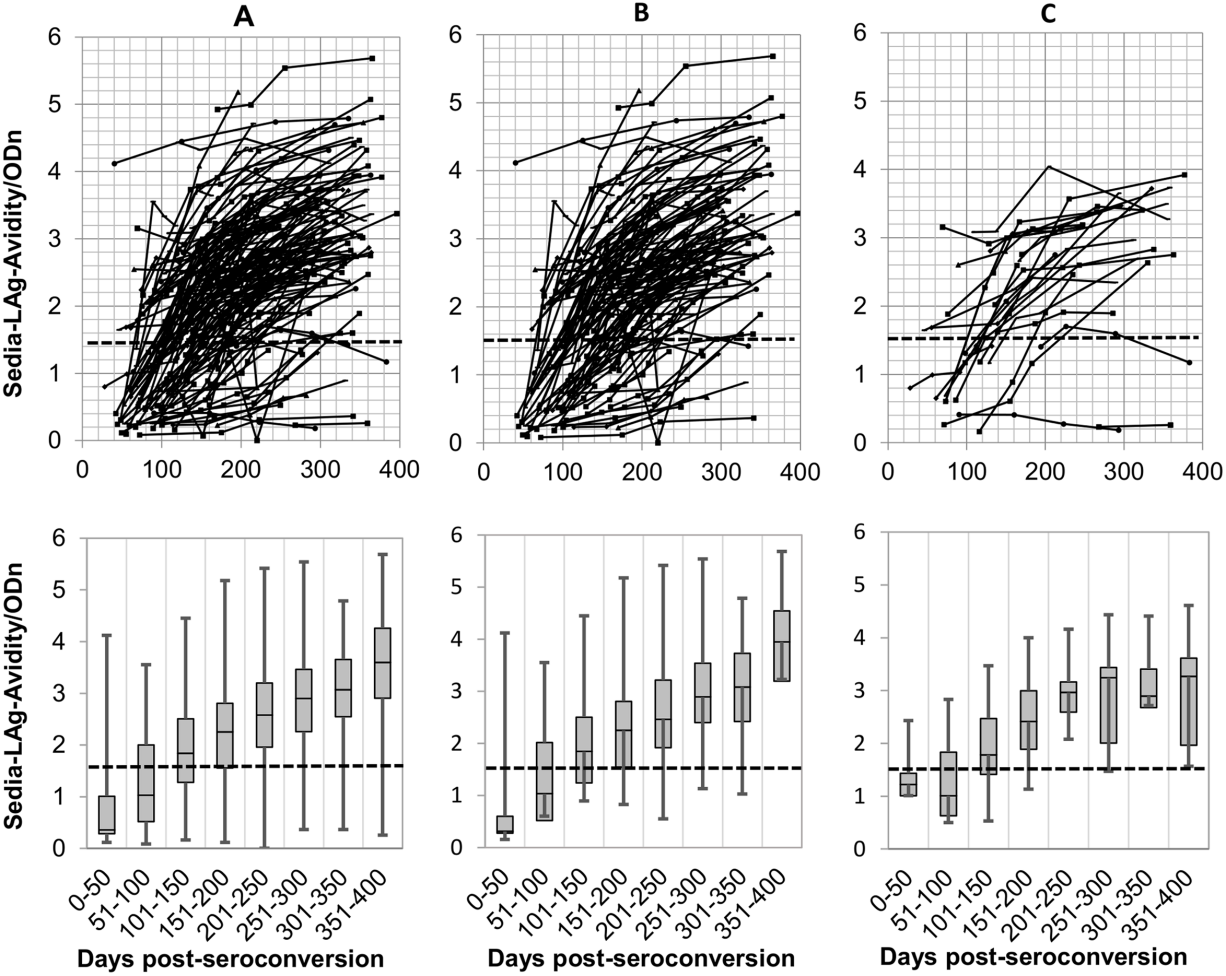
HIV antibody kinetics (up) and box-and-whisker plots showing median of ODn measurements at 50 days intervals (bottom) of the all 150 seroconverting individuals of the PHI cohort of Québec (A), the 123 treatment-naive patients of the PHI cohort of Québec (B), the individuals of the PHI cohort of Québec treated between 0 and 400 days post-seroconversion (C), using the Sedia-LAg-Avidity assay. The ‘recent/long-standing’ infection cutoff value is shown by the horizontal dashed line. ODn: normalized optical density.

Kinetics of antibody avidity development among the 150 seroconverted individuals from the longitudinal PHI cohort (123 treatment-naive and 27 treatment-experienced), as measured by the CDC-modified-Bio-Rad-Avidity and the Sedia-LAg-Avidity assays, are shown in Figs 1 and 2. Using the recommended AI cutoff of 30%, the CDC-modified-Bio-Rad Avidity assay detected more recent infection cases (n = 134) than the Sedia-LAg-Avidity assay (n = 77) at the cutoff of 1.5 ODn, during the first 6 months post infection. Furthermore, the antibody avidity kinetic generated by using the CDC-modified-Bio-Rad-Avidity assay slowly ramped up, as compared to the Sedia-LAg-Avidity assay, resulting in more individuals scattered AI below 30% after one year post-infection, Figs 1A, 1B, 2A and 2B). Conversely, antibody avidity increased over time following a more coherent kinetic using the Sedia-LAg-Avidity assay and fewer individuals were classified below the recommended cutoff of 1.5 after one year post infection. Nine individuals with viral load lower than 2.6 log_10_ copies/mL who were treatment-naive and showed no avidity maturation using both avidity assays.

During the first year post infection, 84 of the 150 MDRI seroconverters did not reach the 30% cutoff with the CDC-modified-Bio-Rad-Avidity assay. Among these 84 individuals who demonstrated a recent infection status, 2/2, 5/8 and 9/17 received ART at 0–30, 91–170, and 171–365, respectively; and 68/84 were ART naive. The number of individuals who did not reach the cutoff of 1.5 ODn with Sedia-LAg-Avidity assay during the first year post infection was lower (16/150); 2/2, 1/8 and 0/17 received ART at 0–30, 91–170, and 171–365, respectively. Thirteen of the 16, were ART naive.

In general, individuals who were exposed to ART during the study expressed avidity maturation profiles similar to those who were ART naive, Figs 1B, 1C, 2B and 2C).

### Performance characteristics of the CDC-modified Bio-Rad-Avidity and Sedia-LAg-Avidity assays using the Quebec Primary HIV-1 Infection Cohort

The performance characteristics of both avidity assays were similar in all and treatment-naive individuals of the PHI cohort. Treatment did not affect the performance characteristics of both avidity assays, Table 2.

**Table 2:** Performance characteristics of the CDC-modified Bio-Rad-Avidity (AI: 30%) and the Sedia-LAg-Avidity (ODn: 1.5) assays.

	Bio-Rad-Avidity				Sedia-LAg-Avidity			
	Treatment-naïve individuals		All individuals		Treatment-naïve individuals		All individuals	
	samples tested; n	‘recent infections’; n (%)	samples tested; n	‘recent infections’; n (%)	samples tested; n	‘recent infections’; n (%)	samples tested; n	‘recent infections’; n (%)
**days post seroconversion**								
<100 (%)	54	52 (96.3%)	71	68 (95.8%)	54	37 (68.5%)	71	48 (67.6%)
100-<200 (%)	219	184 (84.0%)	264	219 (83.0%)	219	62 (28.3%)	264	74 (28.0%)
200-<300 (%)	136	96 (70.6%)	164	113 (68.9%)	136	19 (14.0%)	164	22 (13.4%)
300-<400 (%)	64	30 (46.9%)	78	36 (46.2%)	64	3 (4.7%)	78	5 (6.4%)
MDRI, days [95% CI range]	473	234 [220–249]	577	233 [220–245]	473	120 [109–132]	577	117 [107–130]
FRR (%) [95% CI range]	420	10.2% [7.5–13.5%]			420	6.0% [3.9–8.7%]		

Abbreviations. MDRI: mean duration of recent infection; FRR: false recent rate; CI: confidence interval.

The proportion of individuals classified as ‘recent’ using the CDC-modified-Bio-Rad Avidity assay was higher than that for the Sedia-LAg-Avidity assay for the period of 40 to 200 days post seroconversion. Using the CDC-modified-Bio-Rad-Avidity assay, this proportion decreased slowly over time. In contrary, the proportion of individuals classified as ‘recent’ was decreasing more rapidly using the Sedia-LAg-Avidity assay (<100 days post seroconversion: 67.6%/68.5%; 100–199 days post seroconversion: 28.0%/28.3%; 200–299 days post seroconversion: 13.4%/14.0% and 300–399 days post seroconversion: 6.4%/4.7%) in all individuals *versus* treatment-naive individuals of the PHI cohort, respectively. The MDRI estimate for treatment-naive individuals from the PHI cohort was 234 days and 120 days using the CDC-modified Bio-Rad-Avidity and the Sedia-LAg-Avidity assays, respectively. Similar MDRI estimates (233 days for CDC-modified Bio-Rad- and 117 days for Sedia-LAg-Avidity assays) were obtained when including all PHI cohort individuals.

A total of 10% and 6% (FRR) of long-standing specimens were classified as ‘recent infections’ by using the CDC-modified Bio-Rad-Avidity assay and the Sedia-LAg-Avidity assay.

### Performance characteristics of the Multi-Assay Algorithm (MAA)

To avoid any potential sample misclassification, the performance of multiple assay algorithms (MAA) that use the CDC-modified Bio-Rad-Avidity and the Sedia-LAg-Avidity assays, sequentially, at different index/ODn cutoffs, was evaluated using the 123 treatment-naive individuals from the PHI cohort. The proportion of samples classified as recent by 10 distinct MAAs with avidity cutoff ranges varying from 25% to 40% for the Bio-Rad assay and 1.1 to 2.0 ODn for the LAg assay is shown in Table 3. The performance characteristics of these 10 MAAs in terms of MDRI and FRR are presented in Table 4. The MAA_1_ provided the best FRR (2.14%) and a low MDRI (102 days). However, using MAA_1_, only 30 of 54 samples from individuals infected within 100 days (56%), and 36 of 219 samples from individuals infected between 100 and 200 days (16%), were classified as recent by MAA_1_. On the other hand, MAA_10_ exhibited the highest FRR (above 5%). Using MAA_10_, 40 of the 54 samples from individuals infected within 100 days (74%) and 96 of 219 samples from individuals infected between 100 and 200 days 44%), were classified as recent. The MAAs 4–9 exhibited similar performances with a FRR between 3 and 4% and a MDRI around four months.

**Table 3:** Proportion of samples classified ‘recent’ by different multi-assay algorithms (MAA) including the CDC-modified Bio-Rad-Avidity and the Sedia-LAg-Avidity assays, sequentially.

MAA (AI/ODn)		MAA_1_	MAA_2_	MAA_3_	MAA_4_	MAA_5_	MAA_6_	MAA_7_	MAA_8_	MAA_9_	MAA_10_
		(25/1.1)	(25/1.7)	(25/2.0)	(30/1.5)	(30/1.7)	(35/1.6)	(35/1.7)	(40/1.4)	(40/1.6)	(40/2.0)
Days post seroconversion	Samples tested; N	Samples classified ‘Recent’, n (%)									
40-<100	54	30 (55.6%)	38 (70.4%)	40 (74.1%)	37 (68.5%)	38 (70.4%)	37 (68.5%)	38 (70.4%)	37 (68.5%)	37 (68.5%)	40 (74.1%)
100-<200	219	36 (16.4%)	75 (34.3%)	92 (42.0%)	60 (27.4%)	79 (36.1%)	69 (31.5%)	79 (36.1%)	51 (46.8%)	69 (31.5%)	96 (43.8%)
200-<300	136	13 (9.6%)	22 (16.2%)	29 (21.3%)	18 (13.2%)	22 (16.2%)	21 (15.4%)	23 (16.9%)	19 (14.0%)	21 (15.4%)	30 (22.1%)
300-<400	64	1 (1.6%)	2 (3.1%)	3 (4.7%)	1 (1.6%)	3 (4.7%)	3 (4.7%)	4 (6.3%)	2 (3.1%)	3 (4.7%)	5 (7.8%)

Abbreviations. MAA: multi-assay algorithm; AI/ODn: avidity index %/normalized optical density.

**Table 4:** Performance characteristics of multi-assay algorithms (MAA) including the CDC-modified Bio-Rad-Avidity and the Sedia-LAg-Avidity assays, sequentially, for the treatment naive PHI cohort individuals.

MAA	AI/ODn	MDRI (days)	OR (95% CI)	FRR (%)	OR (95% CI)
MAA_1_	25/1.1	102	92–113	2.1	1.0–4.0
MAA_2_	25/1.7	134	122–146	3.1	1.6–5.3
MAA_3_	25/2.0	144	133–155	4.3	2.5–6.7
MAA_4_	30/1.5	119	108–131	3.3	1.8–5.6
MAA_5_	30/1.7	136	123–148	3.3	1.8–5.6
MAA_6_	35/1.6	129	115–142	3.6	2.0–5.9
MAA_7_	35/1.7	136	124–149	3.8	2.2–6.1
MAA_8_	40/1.4	114	104–127	3.1	1.6–5.3
MAA_9_	40/1.6	129	115–142	3.6	2.0–5.9
MAA_10_	40/2.0	147	136–158	5.2	3.3–7.8

Abbreviations. MDRI; mean duration of recent infection, FRR: false recent rate; CI: confidence interval; AI: avidity index; ODn: normalized optical density; OR: odds ratio.

## Discussion

This is the first study in Canada evaluating the performance of incidence assays assessing the most recently recommended avidity tests, the CDC-modified Bio-Rad-Avidity and the Sedia-LAg-Avidity assays using longitudinal specimens from the Quebec PHI cohort [17, 19, 30]. This cohort consisted mainly of individuals infected with HIV-1 subtype B (99%). Although this type of cohort may exhibit a selection bias, persons seeking medical attention and may not represent the entire HIV epidemics, it remains a gold standard for determining the MDRI as it provides sequential specimens of well-characterized recently infected individuals. Indeed, the nonparametric method analysis we used to estimate the MDRI using the Sedia-LAg-Avidity assay alone gave similar results, 120 days (95% CI; 109–132), than the one obtained by Kassanjee *et al*. [18] and Duong *et al*. [29] who used a binomial regression method analysis. In these studies, the MDRI was estimated at 153 days and 130 days, in a population of patients infected with HIV-1 subtype B, respectively.

Our results showed that the Sedia-LAg-Avidity assay used alone, at the recommended cutoff of 1.5 ODn, misclassified 6% of long-standing specimens as recent infection. Using the CDC-modified Bio-Rad-Avidity assay at the avidity index cutoff of 30%, the rate of long-standing specimens misclassified as ‘recent’ was 10%. Recent studies described much lower FRR 1.6% [29], 0.5% [18]. The higher rates of misclassified long-standing specimens we obtained may be attributed to difference in patient populations used to estimate FRR as time since infection distribution may affect FRR, as recently shown by Patterson-Lomba *et al*.[31]. Indeed, we observed a difference in FRR amongst our own cohorts as specimens that were collected between 14 and 24 months post-seroconversion exhibited a FRR of 13% with the Sedia-LAg-Avidity assay as compared to those collected between 24 months and 19 years which showed a FRR of 4% (data not shown).

The proportion of individuals classified as ‘recent’ was higher using the CDC-modified Bio-Rad-Avidity as compared to the Sedia- LAg-Avidity assay for the period < 200 days post seroconversion.

Therefore, we proposed to start the MAA with the CDC-modified Bio-Rad-Avidity assay. By using such 2-assay algorithm, we are decreasing the rate of false recent infections, as previously shown by Konikoff *et al*. (2013) whose Bio-Rad-Avidity assay protocol differs from the CDC one in the time of incubation of specimens as well as using water to dilute DEA instead of wash buffer [22].

Amongst our treatment-naive study population, approximately 0.8% exhibited CD4^+^ T-cell count below 200 cells/mm^3^ and 7.3% had viral loads below 400 copies/ml, two manufacturer’s prerequisite for the use of LAg-Avidity assay [27]. Therefore adding these parameters did not significantly alter our analysis. This is consistent with a previous study [22] establishing that similar MAA including both avidity assays performed equally for estimating incidence in the presence or the absence of viral load and/or CD4^+^ T-cell count as additional parameters.

Some individuals exhibited high avidity early after infection (Figs 1 and 2). However, others presented with a persistent low avidity suggesting that MAA is better suited for HIV surveillance than for clinical purposes. Interestingly, our study showed that the 27 out of 150 participants of the PHI cohort who were exposed to ART expressed similar avidity profiles to those who were ART naive (123/150), except for 2 seroconverting individuals engaged in very early antiretroviral therapy (0–30 days post seroconversion). For one of these two patients, serial specimens were drawn within 112 days since infection. For the other patient who also exhibited a VL > 2.6 log_10_ copies/mL, the ARV treatment by itself may have been responsible for recency misclassification. Previous studies demonstrated that in patient with recent seroconversion, early antiretroviral therapy may impair antibody production [32, 33] or avidity [33].

The performance of the distinct MAAs for identifying recent infection was described using treatment-naive individuals from the longitudinal PHI cohort. We found that algorithms that include multiple assays were more robust than those with single assays. In addition, the distribution of the times of infection affects the overall performance of incidence assays and MAAs in our population. After evaluation of several MAAs, we observed that the MAA_5_, that uses a CDC-modified Bio-Rad-Avidity cutoff at 30% followed by the Sedia-LAg-Avidity assay at a cutoff of 1.7, provides a greatest power to increase the identification of individuals infected within 5 months and to reduce the rate of FRR. Using this MAA, the MDRI was 136 and the FRR, 3.33%.

We estimate that the use of a MAA has the potential to significantly improve the HIV surveillance program in the province of Quebec, by helping in distinguishing between recent and long-standing infections in newly HIV diagnosed individuals.

## Supporting Information

S1 Table Spreadsheet with Sedia-LAg-Avidity and CDC-modified Bio-Rad-Avidity data (Quebec Primary HIV-1 Infection Cohort) for MDRI estimation. N = 123 treatment-naive and 27 treatment-experienced patients for a total of 577 specimens.
(XLSX)

S2 Table Spreadsheet with Sedia-LAg-Avidity and CDC-modified Bio-Rad-Avidity data for False Recent Rate (FRR) classification. (420 specimens for known long-term infections; 90 from the Quebec Primary HIV-1 Infection Cohort (>14 months post-seroconversion) and 330 from the LSPQ serobank: 2 years post-seroconversion.
(XLSX)
